# Impact and Lessons Learned from Mass Drug Administrations of Malaria Chemoprevention during the Ebola Outbreak in Monrovia, Liberia, 2014

**DOI:** 10.1371/journal.pone.0161311

**Published:** 2016-08-31

**Authors:** Anna Kuehne, Amanda Tiffany, Estrella Lasry, Michel Janssens, Clement Besse, Chibuzo Okonta, Kwabena Larbi, Alfred C. Pah, Kostas Danis, Klaudia Porten

**Affiliations:** 1 Postgraduate Training for Applied Epidemiology, Robert Koch Institute, Berlin, Germany; 2 European Programme for Intervention Epidemiology Training (EPIET), ECDC, Stockholm, Sweden; 3 Epicentre, Paris, France; 4 Epicentre, Geneva, Switzerland; 5 Médecins Sans Frontières, Operational Centre Paris, Tropical Medicine, New York, New York, United States of America; 6 Médecins Sans Frontières, Operational Centre Paris, Monrovia Project, Monrovia, Liberia; 7 Médecins Sans Frontières, Operational Centre Paris, Desk Urgence, Paris, France; 8 Liberian National Malaria Control Programme, Ministry of Health, Monrovia, Liberia; 9 Institut Veille Sanitaire, Paris, France; Centers for Disease Control and Prevention, UNITED STATES

## Abstract

**Background:**

In October 2014, during the Ebola outbreak in Liberia healthcare services were limited while malaria transmission continued. Médecins Sans Frontières (MSF) implemented a mass drug administration (MDA) of malaria chemoprevention (CP) in Monrovia to reduce malaria-associated morbidity. In order to inform future interventions, we described the scale of the MDA, evaluated its acceptance and estimated the effectiveness.

**Methods:**

MSF carried out two rounds of MDA with artesunate/amodiaquine (ASAQ) targeting four neighbourhoods of Monrovia (October to December 2014). We systematically selected households in the distribution area and administered standardized questionnaires. We calculated incidence ratios (IR) of side effects using poisson regression and compared self-reported fever risk differences (RD) pre- and post-MDA using a z-test.

**Findings:**

In total, 1,259,699 courses of ASAQ-CP were distributed. All households surveyed (n = 222; 1233 household members) attended the MDA in round 1 (r1) and 96% in round 2 (r2) (212/222 households; 1,154 household members). 52% (643/1233) initiated ASAQ-CP in r1 and 22% (256/1154) in r2. Of those not initiating ASAQ-CP, 29% (172/590) saved it for later in r1, 47% (423/898) in r2. Experiencing side effects in r1 was not associated with ASAQ-CP initiation in r2 (IR 1.0, 95%CI 0.49–2.1). The incidence of self-reported fever decreased from 4.2% (52/1229) in the month prior to r1 to 1.5% (18/1229) after r1 (p<0.001) and decrease was larger among household members completing ASAQ-CP (RD = 4.9%) compared to those not initiating ASAQ-CP (RD = 0.6%) in r1 (p<0.001).

**Conclusions:**

The reduction in self-reported fever cases following the intervention suggests that MDAs may be effective in reducing cases of fever during Ebola outbreaks. Despite high coverage, initiation of ASAQ-CP was low. Combining MDAs with longer term interventions to prevent malaria and to improve access to healthcare may reduce both the incidence of malaria and the proportion of respondents saving their treatment for future malaria episodes.

## Introduction

In 2014 and 2015 the largest Ebola outbreak in history occurred in West Africa with more than 28,000 confirmed, probable and suspect Ebola cases reported in three countries with intense transmission–Guinea, Sierra Leone and Liberia [1]. Between March 2014 and July 2015, more than 10,500 suspect Ebola cases were notified in LiberiaThe majority of cases and deaths were identified in Montserrado County where the capital city Monrovia is located [2].

As a consequence of the Ebola outbreak existing healthcare structures in Monrovia were rapidly overwhelmed. By October 2014 few health facilities in Monrovia were fully operational and those that were open seemed to offer limited services. Laboratory testing for any medical condition, including rapid diagnostic tests (RDTs) for malaria was largely unavailable outside Ebola treatment units (ETUs) and Liberian National Malaria Control Program (NMCP) recommended presumptive malaria treatment.

At the same time, malaria, the main killer of children under five years of age and among the most common causes of outpatient consultations and inpatient deaths in Liberia [3, 4], was circulating in the community. Malaria is endemic in Liberia and despite a seasonal peak in July [5], transmission occurs year round [4]. In October 2011, microscopy confirmed malaria prevalence was estimated to be 7% in children under 5 years of age in Monrovia [4]. Since 2003, artesunate/amodiaquine (ASAQ) is the recommended first-line treatment for uncomplicated malaria in Liberia [4, 6].

Clinical presentation of Ebola and malaria cases is similar and differentiation without laboratory tests often impossible. In Liberia the case definition used for suspect Ebola cases (fever plus three unspecific symptoms) was highly sensitive in order to capture all Ebola cases. However, it also captured patients with malaria. As a result, an unknown number of malaria patients remained either unattended to due to the collapse of the healthcare system or were admitted to an ETU as a suspect Ebola case. Consequently, malaria patients were put at risk of exposure to Ebola and ETU resources were further strained by the admission of substantial numbers of non-Ebola patients. While the number of confirmed Ebola cases in Monrovia began to decline in October 2014 [7], malaria transmission continued and healthcare for non-Ebola-illnesses including malaria remained limited (Médecins Sans Frontières unpublished operational data). Thus, Médecins Sans Frontières (MSF) initiated mass administrations of antimalarial drugs in cooperation with the Liberian NMCP in four administrative zones of Monrovia, to decrease malaria-associated morbidity and mortality in order to mitigate the reduced access to general healthcare and reduce admissions to ETUs for malaria-associated fever.

The administration of therapeutic antimalarial regimens to entire populations regardless of clinical symptoms or laboratory tests has been part of malaria control programs for more than a century [8, 9]. After constituting a component of many malaria elimination programs in the 1950s, the World Health Organisation (WHO) stopped recommending MDAs due to uncertainty about their ability to interrupt transmission and concerns about their potential to increase drug resistance [8, 10]. However, evidence accumulated indicating that drug regimens, specifically those including artemisinin-based combination therapies (ACT) in therapeutic doses, significantly reduced parasitaemia prevalence as well as malaria morbidity and mortality over time [8, 10, 11]. In November 2014, WHO recommended MDAs of ACTs regardless of malaria symptoms for the countries affected by the Ebola outbreak in West Africa in areas with high Ebola morbidity, high malaria transmission and limited access to treatment [12].

Mass administrations of antimalarial drugs to reduce the number of fever cases during an Ebola outbreak had never been carried out before. We aimed to describe the scale of the MDA, to evaluate community acceptance of the strategy, adherence to treatment, and estimate the effectiveness of the intervention with regards to the reduction of fever cases in order to inform future public health interventions targeting fever reduction in an Ebola context.

## Methods

### Design and implementation of the MDA

**Fig 1:**
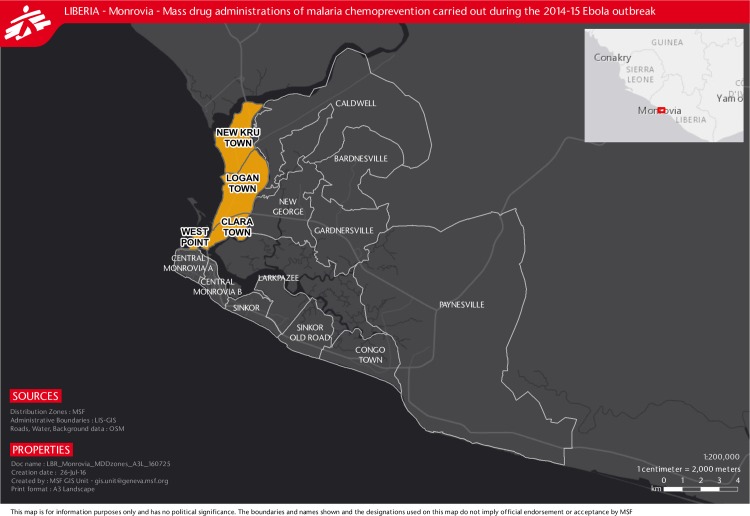
Areas of mass drug administrations (MDA) of artesunate/amodiaquine malaria chemoprevention (ASAQ-CP) during the Ebola outbreak, Monrovia, Liberia, 2014.

Four zones in Monrovia were targeted for the MDA based on their high Ebola incidence, high population density, precarious living conditions and limited access to healthcare. The population of these zones, New Kru Town, Clara Town, Westpoint and Logan Town (Fig 1), was initially estimated at 300,000 individuals (MSF census in collaboration with community leaders, unpublished programmatic data).

Two rounds of MDA were carried out in a stepwise fashion at 56 fixed points of distribution between October and December 2014 with a one-month interval between rounds. The drug distributed was ASAQ and was specifically procured for the MDA. One course of ASAQ consists of three doses to be taken once a day over three consecutive days in age-specific, fixed-dose-combinations. ASAQ is used as treatment for uncomplicated malaria infections and the post-treatment prophylactic effect is thought to last for at least one month after the full course is taken [13].

**Fig 2:**
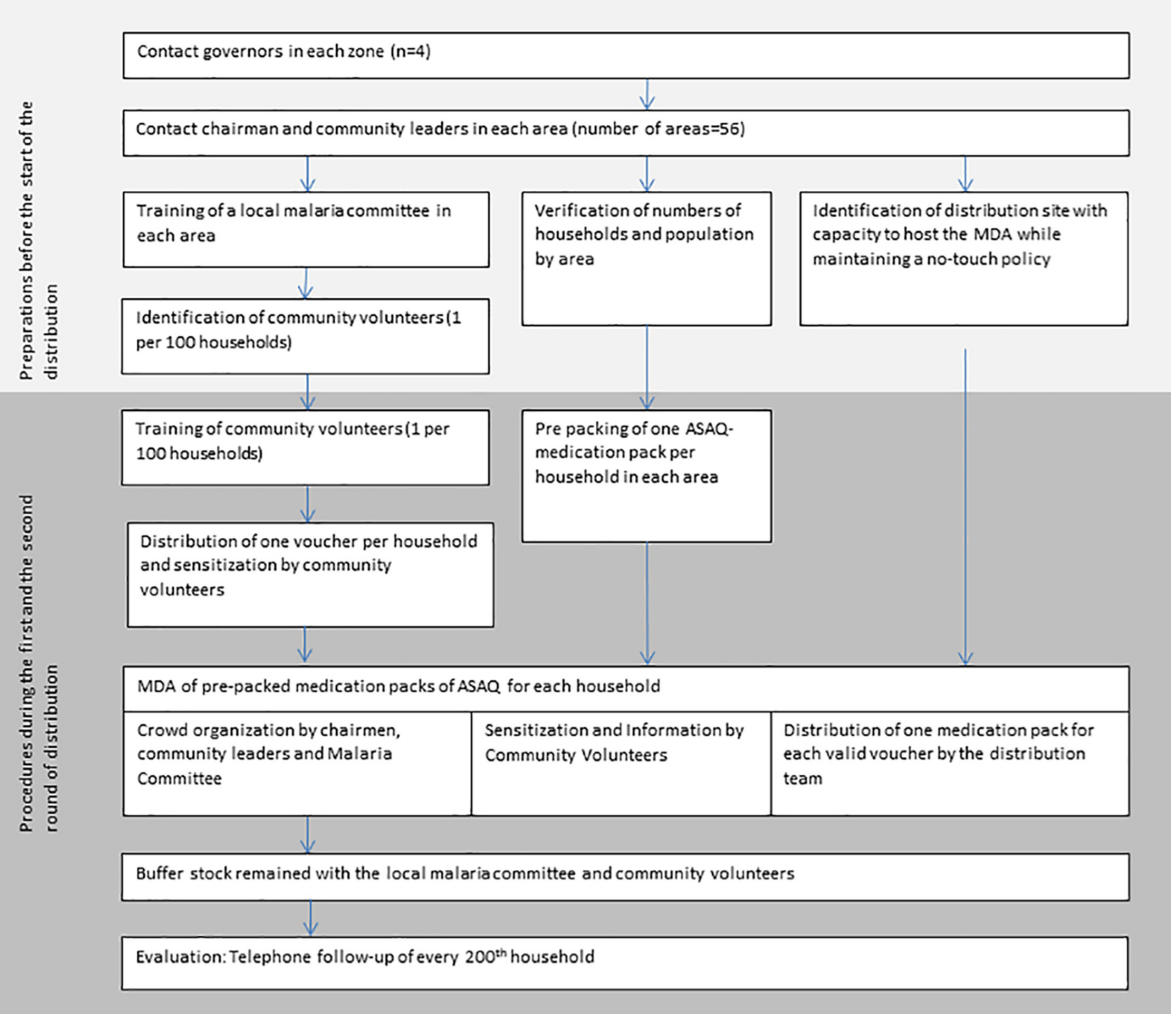
Steps of implementation of the mass drug administration (MDA) of artesunate/amodiaquine malaria chemoprevention (ASAQ-CP) during the Ebola outbreak, Monrovia, Liberia, 2014.

The distribution team met with the governor of each zone prior to the distribution (Fig 2).

After agreement with the governor, the chairmen/chairwomen and community leaders (CCL) of all areas in the zone were contacted by the distribution team. Together with CCL a local malaria committee comprised of numerous community authorities including representatives of minority groups was established and population numbers were verified. In collaboration with the malaria committee, one community volunteer (CV) was identified per 100 households and trained by the distribution team to provide sensitization messages to the population. These messages included the information i) that preventive malaria treatment will be distributed, ii) age-specific doses will be provided, iii) doses should be taken with food, three days in a row. These messages were adapted regularly during the intervention. CVs went door-to-door (without entering the house) the day before the MDA explaining the purpose and content of the MDA to household members and distributing one voucher to each household. The voucher entitled one woman of the household to receive one pre-prepared medication pack for their household, from the designated distribution point, on the day of the MDA.

To avoid crowding, the MDAs were carried out at fixed points in the early morning during curfew for a maximum of two hours. Crowd-management and security were the responsibility of CCL and the malaria committee. Women attending the distribution were asked to stand in lines, CVs were present to re-emphasize sensitization messages and to ensure adherence to the no-touch policy. The set-up of the easy-to-carry and easy-to-disinfect distribution site was organized in a way that avoided crowding and ensured rapid distributions.

On the distribution day, women could exchange the voucher they received from the CV for a pre-prepared medication pack of ASAQ containing a full course of ASAQ-chemoprevention (ASAQ-CP) for seven household members (two adults and five children of various age groups). Children below 6 months did not receive ASAQ-CP. The medication packs were pre-prepared to keep logistics light and to facilitate rapid distribution. A catch-up distribution was carried out if less than 85% of the expected households attended the distribution at the respective site. In addition to the MDA, a back-up stock of medication packs remained with designated members of the community after the MDA. Households that did not receive sufficient ASAQ for all household members had the possibility to collect the missing ASAQ-CP doses.

The distribution team documented the number of vouchers and medication packages distributed by area in each round and the number of necessary catch-up distributions.

### Objectives and design of the evaluation

We used data collected by the distribution team to assess the target population size, the absolute numbers of vouchers and medications packages distributed during the MDA and the number of catch-up distributions carried out.

For further evaluation of the MDA, one in every 200 households that received vouchers in round 1 (r1) of the MDA was randomly selected for telephone follow-up through systematic sampling during voucher distribution.

The objectives of the evaluation were to:

1. Describe the scale of the MDA: We described the absolute numbers of vouchers and medication packs distributed and the number of catch-up distributions.
2. Estimate acceptance of the MDA and adherence to ASAQ: We estimated attendance at the MDA and described the attitude towards the distributions on household level. On individual level, we estimated the availability of ASAQ and compliance with initiation of and adherence to ASAQ-CP and identified reasons for non-compliance and non-adherence. Additionally, we estimated the rate of self-reported side effects and examined the association between side effects and ASAQ-CP initiation.
3. Estimate the effectiveness of MDA: We estimated the incidence of self-reported fever episodes before and after the first round of MDA.

### Procedures and data collection of the evaluation

Every 200^th^ voucher was marked by a triangle shaped punch cut into the plastic voucher. The CVs that carried vouchers informed each household that received a marked voucher about the purpose of the evaluation and asked the head of the household (or another adult, if not available) for consent. If they refused, the next household to whom the CV distributed a voucher was asked for consent. For logistical reasons, no information about households was collected by the CVs during telephone number collection. After consenting, the respondents provided their telephone number. One week after both MDA r1 and r2 had taken place in the area, a trained surveyor interviewed the respondents by telephone using a structured questionnaire. The questionnaires collected information on MDA attendance and acceptance. The respondent provided information for every member of his/her household regarding availability of ASAQ-CP for all household members, initiation and completion of the ASAQ-CP course, side effects and fever in the previous month. A question regarding the availability of the appropriate age-specific dose was added during the first round.

To assess the feasibility of the evaluation and the composition of the medication packs, we conducted a pilot evaluation. This included households from the first five distribution days, approximately 20% of all households. Data from these households was excluded from the evaluation.

### Operational definitions of the evaluation

A household was defined as a group of people living under the same roof and sharing the same meal at least three times a week regardless of family ties.

Self-reported fever was used as a proxy for malaria infection and defined as history of fever or malaria within the past month reported by the respondent. Reports could not be confirmed by laboratory testing due to the ongoing Ebola outbreak.

Reported side effects were defined as any sign or symptom reported as a side effect by the respondent.

Compliance with treatment initiation was defined as taking at least one of the three doses of ASAQ-CP as reported by the respondent.

Adherence was defined as taking all three doses of ASAQ-CP, thus completing the full course as reported by the respondent.

### Data analysis for the evaluation

We described absolute population numbers and MDA attendance and number of catch-up distributions as collected by the distribution team.

For further evaluation, households that were reached for telephone interviews in both rounds of MDA were included in the analysis. As one individual was reporting for his or her whole household in both rounds, individual household members needed to be re-identified in the second round to allow for individual level analysis of data; re-identification was based on reported age and sex as data collection was anonymous and names were not recorded. First we re-identified individuals with identical ages in both rounds. Next, in order to account for imprecise age estimations by household members, we re-identified individuals ± 1 year (or months for infants below 12 months of age) during the second round followed by individuals ±2 years of age. Household members that were only reported in one of the two rounds were not included in the analysis as measures of associations cannot be calculated for them.

On household level, we calculated proportions. On an individual level we calculated proportions with 95% confidence intervals (95% CI) where appropriate, allowing for the potential cluster effect of the household on treatment availability, compliance with treatment initiation and adherence. Differences between household members initiating and adhering to treatment and those that did not were calculated with chi squared tests.

We used poisson regression to calculate incidence ratios (IR) as a measure of association between reported side effects and treatment initiation, adjusting for possible confounders and also taking into account the cluster effect of the household.

As fever was reported for the past month, the evaluation after r1 captured self-reported fever incidence prior to the start of the MDA and the evaluation after r2 captured the self-reported fever incidence the month after r1. We calculated the difference in reported fever by subtracting the incidence of reported fever episodes after r2 from the incidence of reported fever episodes after r1 and presented risk differences (RD) with 95% CI. We compared the reported pre- and post-r1 incidences using a z-test for difference in proportions. Additionally, we compared the RD among household members that took the full course of ASAQ in r1 with the RD among household members that did not take the full course of ASAQ with a z-test for difference in proportions.

Analyses were conducted with STATA version 13 (Stata corporation, Texas, USA).

### Ethical considerations

The data presented here are from an analysis of MSF programmatic data. The procedures conducted during the evaluation were in accordance with the ethical standards of the Helsinki Declaration. The CVs read a consent form to the head of the household and explained that participation was voluntary. Respondents provided verbal consent. No personal identifiers were collected and only aggregated data was reported. All evaluation documents were stored in a locked cabinet or a password protected computer.

## Results

### Scale of the MDA

In the four targeted zones of Monrovia a total population of 551,971 individuals in 102,372 households was verified with CCL and the malaria committee in round 1 and corrected to 558,483 individuals in 103,497 households during r2. 102,372 vouchers for a pre-packaged medication pack were distributed during round 1 and 103,497 during r2. The total number of vouchers exchanged for medication packs was 90,411 (94%) in r1 and 89,546 (93%) in r2. In total, 1,259,699 courses of ASAQ-CP were distributed.

At each of the 56 distribution points in both rounds, between 347 and 3,853 women received medications packs, depending on community size.

Catch-up distributions needed to be carried out in three (5%) of the 56 sites in r1. In r2, two catch-up distributions were deemed necessary but only one was carried out; in the other community, chairmen were reluctant to organize a new distribution as they were convinced that it was not necessary.

### Response

The communities included in the evaluation (excluding the pilot evaluation) consisted of 426,760 individuals in 72,585 households of which every 200^th^ was asked to participate.

We aimed to contact 365 selected households by phone in rounds 1 and 2. CVs reported that every selected household agreed to participate and had a phone. Of the 365 selected households, 222 (61%) were reached by phone in both rounds. The remaining 39% (143/365) of sampled households either i) did not answer their phone, or ii) had their phone turned off, or iii) the connection was too poor to collect any information during three tries. Of the 143 households that were not included in the evaluation, 47% (67/143) were only reached in one of the rounds of the MDA and 53% (76/143) were not reached in any round. All 222 households that were reached in both rounds had provided informed consent to the CV beforehand and agreed to be interviewed.

In total, 1,643 individuals were reported to live in the 222 households reached in both rounds (average household size: 7.4). Using household-code, sex and age, 1,236 (75%; 1236/1643) individuals could be re-identified in both rounds. The 1,236 individuals were principally female (54%, 667/1,236) with a median age of 16 (Interquartile range: 8–28 years). The 407 (25%) individuals who were unable to be re-identified were reported to have lived in the household during only one of the two rounds of MDA.

### Attendance, compliance and adherence

All 222 households attended the distribution during r1 (100%), accounting for 1,236 household members of which 1,233 were eligible for ASAQ-CP (i.e. household members older than six months). During r2, 212 (96%) households attended the MDA, accounting for 1,157 household members of which 1,154 were eligible for ASAQ-CP. Of the eight households that could not attend the MDA in r2, six were not in the distribution area during the distribution of medication packs, one did not receive a voucher and one lost the voucher.

Despite the availability of buffer stocks, some household members did not receive ASAQ-CP. In r11, 1,113 (90%) of 1,233 household members received sufficient ASAQ-CP, compared with 1,144 (99%) of 1,154 in r2 (Table 1).

**Table 1:** Reported compliance and adherence with artesunate/amodiaquine malaria chemoprevention (ASAQ-CP) among households targeted for mass drug administration (MDA) during the Ebola outbreak, Monrovia, Liberia, 2014.

Response for all household members		Round 1 of MDA (N = 1233ᶲ)			Round 2 of MDA (N = 1154ᶲ)		
		n	% of N	(95% CI)	n	% of N	(95% CI)
**Household members who received sufficient ASAQ-CP**	Unknown	48	3.9	(1.5; 9.8)	0	0.0	-
	No	72	5.8	(3.7; 9.0)	10	0.9	(0.3; 2.3)
	Yes	1113	90	(85; 94)	1144	99	(98; 100)
**Household members who initiated ASAQ-CP**	Unknown	8	0.7	(0.3; 1.6)	0	0.0	-
	No	462	38	(32; 43)	888	77	(71; 82)
	Yes	643	52	(46; 58)	256	22	(17; 28)
Household members who adhered to the full course of ASAQ-CP*	Unknown	0	0.0	-	0	0.0	-
	No	51	4.1	(2.3; 7.2)	3	0.3	(0.1; 1.3)
	Yes	592	48	(42; 54)	253	21	(17; 28)

ᶲ 3 infants < 6 months were not included in the analysis

* includes household members that reported having just started the 3-day-course of chemoprevention and planning to complete the course of ASAQ-CP within the next two days

Among those household members that received any dose, the dose received was reported to be age appropriate for ≥98% of household members in both rounds. During r1, 98% (983/1,005) of household members for whom information was available, reported receiving the correct dosage of ASAQ-CP for their age, 21 (2%) did not know, and one (0.1%) reported receiving an incorrect dose. In r2, 100% (1,144) household members reported receiving the correct dose.

Compliance with ASAQ-CP initiation among those that attended the MDA was 52% (643/1233) in r1 and 22% (256/1154) in r2 (**Table** 1). There were no significant differences in age or gender between individuals who did and did not initiate ASAQ-CP in r1 and r2: In r1 44% of household members initiating ASAQ-CP were male, median age was 15 years. 47% of household members not initiating ASAQ-CP were male and median age was 17 years. In r2 44% of household members initiating ASAQ-CP were male, median age was 15 years. 45% of household members not initiating ASAQ-CP were male and median age was 16.5 years.

In r1 48% (592/1233) of all household members that received ASAQ-CP adhered to the full course, in r2 21% (253/1154) (**Table** 1). In r1, 51 individuals did not complete their treatment; 22 (43%) were male; median age 15 years. In r2 only 3 individuals did not complete their treatment; 1 (33%) was male; median age 10 years.

### Community acceptance of the MDA and reasons for non-compliance and -adherence

After r1 all but one household approved of the way the distribution was carried out; the disapproving respondent mentioned that some people arrived with multiple vouchers and got more than one household-pack of ASAQ-CP. No complaints were reported after r2.

**Fig 3:**
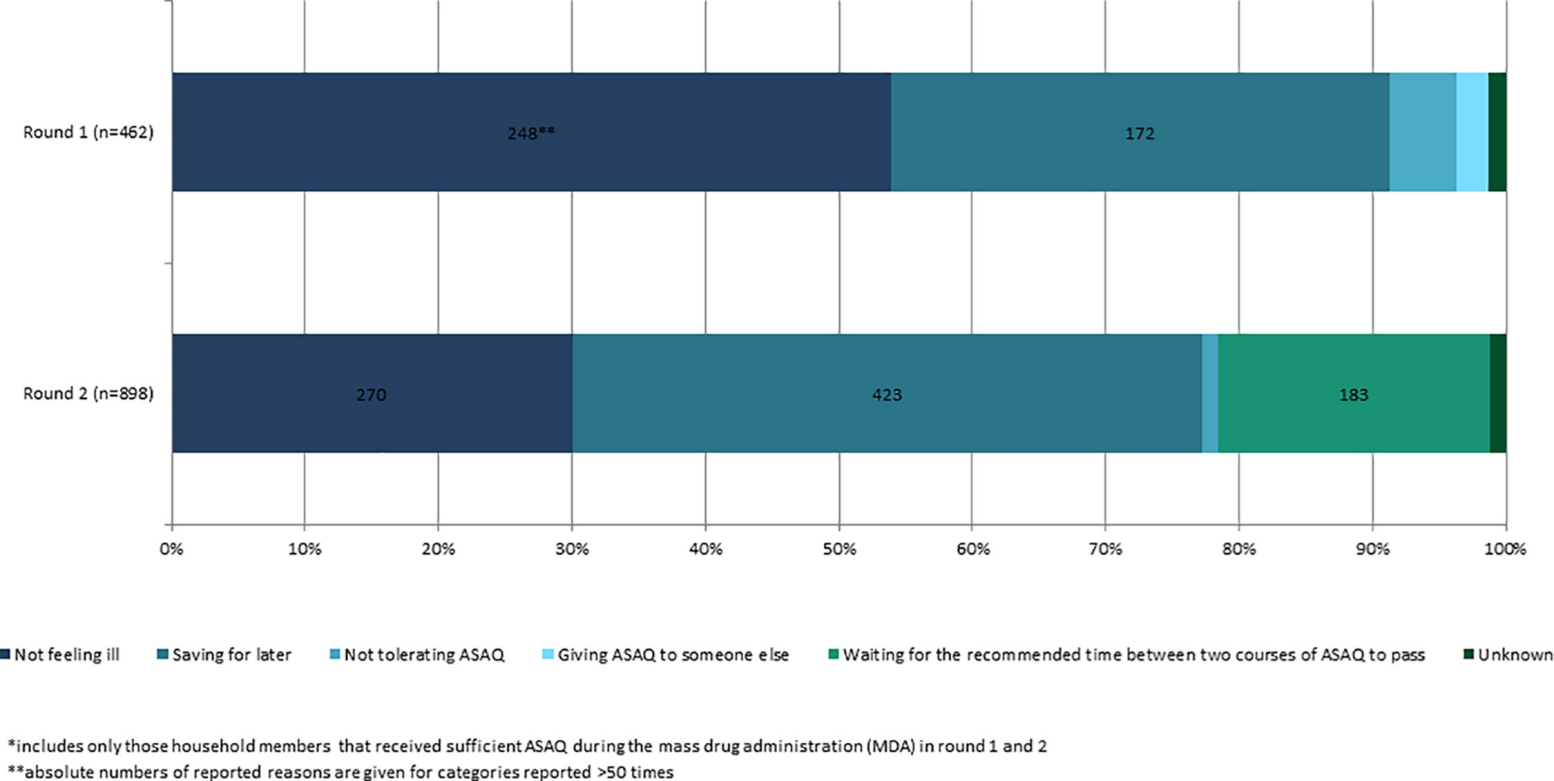
Reported reasons for non-compliance with initiation of artesunate/amodiaquine malaria chemoprevention (ASAQ-CP) during the Ebola outbreak, Monrovia, Liberia, 2014*.

In r1, 248 (54%) of 462 household members not initiating the course of ASAQ-CP reported they did not feel sick, and 172 (37%) that they had saved ASAQ for later. In r2, 423 (48%) of 888 not compliant with ASAQ-CP initiation reported saving it for later, 270 (30%) reported not feeling sick and 183 (21%) that they waited for one month between the two rounds of chemoprevention (Fig 3).

Adherence to the three day ASAQ-CP course among those who initiated ASAQ-CP increased from 91% (592/643) in r1 to 99% (253/256) in r2 of MDA; those completing ASAQ-CP and those not completing ASAQ-CP did not differ in age and gender. In r1, 14 household members reported side effects as the reason for interruption, 12 did not understand the importance of completing a full course, eight shared their course with someone else and 17 reported other or unknown reasons. In r2, three household members did not complete the ASAQ-CP course; one reported side effects as the reason and two reported not understanding the importance of continuing the three-day course. Among the 605 household members who initiated ASAQ-CP in r1 and attended the MDA in r2, 406 (67%) decided not to initiate ASAQ-CP in the second round. The most frequently reported reasons were saving ASAQ for later (49%; 198/406), waiting for the recommended time between two ASAQ courses to pass (25%; 100/406) and not feeling ill (24%; 96/406).

### Side effects of ASAQ and their impact on adherence during the MDA

Reported side effects among household members starting ASAQ-CP in each round included: drowsiness (r1: 10%; r2: 6%), dizziness (r1: 10%; r2: 5%), fever (r1: 2%; r2: 0.0%), nausea (r1: 1%; r2: 0.0%), headache (r1: 1%; r2: 0.0%), vomiting (r1: 1%; r2: 0.4%) and skin reactions (r1: 0.6%; r2: 0.0%). Overall attack rate of any reported side effect in the first and second round of MDA was 17% (107/643) and 6% (16/256), respectively. Among those household members that started ASAQ-CP, 15 (1.7%) of 899 ASAQ-CP courses during both rounds of MDA were reported to be interrupted for possible ASAQ side effects.

In r1, 23 (2%) of 1,113 household members that received sufficient ASAQ reported that they did not initiate ASAQ-CP because they feared side effects. In r2, 11 (1%) of 1,144 household members did not initiate due to ASAQ side effects. Yet, occurrence of side effects during r1 was not associated with ASAQ-CP initiation during r2 when adjusted for age and gender (Table 2).

**Table 2:** Association between experiencing side effects of artesunate/amodiaquine malaria chemoprevention (ASAQ-CP) in the first round of mass drug administration (MDA) with initiation of ASAQ-CP in the second round of the MDA, during the Ebola outbreak, Monrovia, Liberia, 2014 (Poisson regression).

Characteristics of household members*	(N = 591)	Adjusted IR	(95% CI)	p-value
**Household members reported experiencing side effects in round 1**				
No	487	reference		
Yes	104	1.00	(0.61; 1.64)	0.99
**Age**				
Age in years	591	1.00	(0.99; 1.01)	0.40
**Gender**				
Male	258	reference		
Female	333	0.97	(0.77; 1.22)	0.76

* includes only household members that initiated ASAQ-CP in round 1 and received ASAQ-CP in round 2

Among household members that initiated ASAQ-CP in r1 and provided information on side effects in r1 and ASAQ-initiation in r2, the proportion of household members initiating ASAQ-CP in r2 was 32%, both among those that experienced side effects (33/104) and among those that did not experience side effects (158/487).

### Effectiveness of the MDA

The incidence of self-reported fever episodes decreased significantly after r1 compared to the month prior to r1, from 4.2% to 1.5% (p<0.0001) (Table 3).

**Table 3:** Incidence and risk difference (RD) of self-reported fever episodes among household members that attended the mass drug administration (MDA) of artesunate/amodiaquine malaria chemoprevention (ASAQ-CP) in rounds 1 and 2 during the Ebola outbreak, Monrovia, Liberia, 2014.

Responses for all household members		Incidence of self-reported fever episodes (%) in the month prior to round 1	Incidence of self-reported fever episodes (%) in the month prior to round 2	RD (%) of self-reported fever episodes	(95% CI for RD)	p-value
All household members (N = 1229ᶲ)		4.2	1.5	2.7	(1.4; 4.0)	<0.001*
**Incidence of self-reported fever by ASAQ-CP adherence**						
	Household members who completed ASAQ-CP in round 1 (N = 592^‡^)	6.4	1.5	4.9	(2.7; 7.1)	<0.001*
	Household members who did not start or complete ASAQ-CP in round 1 (N = 511^‡^)	2.2	1.6	0.6	(-1.1; 2.2)	0.690
**Incidence of self-reported fever by age group**						
	Household members >5 years old (N = 1044)	3.8	1.6	2.2	(0.8; 3.6)	0.002*
	Household members < = 5 years old (N = 185)	6.5	1.1	5.4	(1.6; 9.2)	0.006*

ᶲ 6 household members for whom incidence of self-reported fever is unknown were excluded

^‡^ Further 126 household members for whom compliance with ASAQ-CP initiation or adherence was unknown were excluded from analysis

* Significant difference in incidence of self-reported fever between round 1 and 2 of the MDA

Reported fever incidence was initially higher in children than in adults: self-reported fever incidence was 6.9% in children ≤ 5 years of age and 3.8% in older household members and decreased to 1.1% and 1.6% respectively after the first round of the MDA. Incidence of self-reported fever was 4.9% lower after r1 among those household members who took a full course of ASAQ-CP and 0.6% lower among household members who did not start or not complete a full course of ASAQ-CP (Table 3). While reported incidence decreased in both groups, the RD was significantly bigger among the group that took the full course (p<0.001).

## Discussion

The MSF-lead MDA in Monrovia was a novelty and a challenge as MDAs have rarely been carried out on such large scale, never with ASAQ [9, 11] and for the first time during an Ebola outbreak. Several challenges were encountered and lessons learned from the MDA which could help to inform future interventions (Box 1).

**Box 1. Challenges and lessons learned from the mass drug administration (MDA) of artesunate/amodiaquine malaria chemoprevention (ASAQ-CP) during the Ebola outbreak, Monrovia, Liberia, 2014.**

■ Verification of population estimates with local leaders prior to the distribution substantially increased the estimated population size but was necessary to ensure appropriate coverage.
■ Use of fixed points for distribution of pre-packed medications using stringent infection prevention and control procedures was challenging but fast and feasible.
■ Coordination of messages with relevant actors in the target area is important; adaptation of messages according to monitoring results can improve compliance and adherence.
■ If directly observed treatment (DOT) is impossible, treatment initiation can possibly be improved by distributing familiar and uniformly packed medication and by provision food and ORS at the same time.
■ Occurrence of treatment related side effects did not affect adherence.
■ Adherence to ASAQ-CP was high and no risk of drug resistance development was identified.
■ No amplification of the Ebola outbreak in the target areas was observed during or after the intervention.
■ Self-reported fever cases were significantly lower after the MDA, particularly among household members that initiated ASAQ-CP.
■ Combining MDAs in malaria endemic areas with longer term interventions to prevent malaria and to improve access to healthcare may reduce the proportion of respondents saving their treatment for future malaria episodes.

### Scale and challenges in implementation of the MDA

After discussions with CCL and the malaria committee in all 56 distribution sites the initial population estimate used to plan the distribution increased by 184% (300,000 to 551,971). While a challenge for planning, overcrowding and multiple families using the same shelter in shifts may explain the initial underestimation. An additional increase of approximately 7,000 people was noted from the first to second round and was due to the initial accidental exclusion of several small minority communities. These minority communities were not deemed part of their community by CCLs during planning for r1 and were only discovered by local MSF staff after the first distribution.

Engagement of local staff and close collaboration with both the local CCL and malaria committee ensured one woman per household would attend the distribution. Similar to other distributions where the importance of community engagement was highlighted as critical to ensuring high campaign coverage [9, 11], these relationships proved essential in reaching more than 80% of the population in each target community [11]. Community sensitization was key however challenging. Different actors carried out distributions of various items during the same period in Monrovia with little coordination. Consequently, CVs, depending on the organization for which they were working, provided different sensitization messages and announced different distribution schedules and places. Additionally, the distributions took place during a period when trust in international aid and the Ebola response was not high and rumours about the spread of Ebola by international actors were common.

Compliance with ASAQ-CP initiation may have been improved by utilization of directly observed treatment (DOT), a strategy cited as an essential component of successful MDAs in the past [11]. Indeed, WHO recommended door-to-door distributions with DOT for MDAs during the Ebola outbreak [12]. However, in the most populated areas of Monrovia with the highest Ebola incidence, door-to-door distributions and the DOT strategy were deemed unfeasible due to concerns for staff security and the risk of amplifying Ebola transmission. Use of fixed distribution points allowed us to mitigate the risks of Ebola transmission among staff and beneficiaries through use of rigorous crowd-management and a no-touch-policy. Furthermore, strict infection prevention and control procedures were implemented for distribution sites, staff and cars. These measures would not have been possible to implement during door-to-door distributions. Despite ongoing Ebola transmission in the distribution areas during the MDAs, no Ebola case associated with the MDAs presented at the MSF ETU in Monrovia or was brought to the attention of MSF, NMCP or other actors. Additionally, several Ebola transmission chains ended in the target area during that period. Thus, we identified no risk of fuelling the Ebola outbreak during the MDAs.

### Acceptance, compliance, adherence–Lessons learned

Even though almost 100% of recipients reported a positive attitude towards the MDA, the majority did not initiate ASAQ-CP; most reported not feeling sick and saving ASAQ for later. In other MDA interventions with similar experiences, more than one third of respondents reported being reluctant to take the available drugs because they did not feel sick [11]. In Monrovia, this could be a consequence of national health messaging prior to the Ebola outbreak that emphasized the importance of being tested for malaria before initiating treatment. During the start of the distribution local staff reported that this message continued to be communicated by other actors in parts of the target areas.

Some of the ASAQ doses however, were procured from a manufacturer different to that manufacturer which provided the most commonly available ASAQ drugs in Monrovia prior to the outbreak. This may have led to confusion among recipients as the packaging was different and community members may have been less familiar with the drug name than the packaging itself. Due to issues with supply, the household medication packs were pre-prepared in plastic bags of varying colours. This may have caused some additional confusion among recipients who suspected that the contents of each bag were different based on its colour.

Anecdotal reports from local nurses and CVs highlighted the role that the absence of food and oral rehydration solution (ORS) distributions concurrent with the drug distribution may have had on treatment initiation. Indeed, it was recommended to take ASAQ with food and to use ORS in case of vomiting or diarrhoea–both unavailable to some recipients living in the most precarious areas of Monrovia. Finally, ASAQ-CP may have been saved more frequently during the second round because residents realised that MSF would not be carrying out a third MDA and health facilities had yet to reopen. The risk of future infection with malaria and the lack of available treatment in the continuous absence of healthcare facilities or future MSF distributions may have been perceived as more important. Consequently, the best use of ASAQ would be as treatment when sick.

Although a concern, no evidence has been found in the past that MDAs of full course therapeutic treatment with combination drug regimens lead to increased drug resistance [9–11]. We were able to increase ASAQ-CP adherence to the three-day course from 90% to close to 100% between r1 and r2 thus minimizing the risk of amplification of resistant parasite strains. Increase in ASAQ-CP adherence may be the result of adapted messaging after preliminary analysis of the first round data. For the second round CVs were encouraged to stress the importance of adherence and the availability and location of the buffer stock for those household members who did not receive ASAQ-CP.

### Side effects of ASAQ and their impact on adherence during the MDA

ASAQ was selected as CP due to its availability, proven efficacy [13], safety, tolerability [14] and the communities’ familiarity with it. During this intervention ASAQ-CP led to common but mild side effects that did not affect adherence in the two rounds of MDA. Interruptions of ASAQ-CP course due to reported side effects were rare. These findings are consistent with previous studies of ASAQ-CP related adverse events. Severe adverse events were recorded in only 3% of respondents and none of these led to treatment interruption or long-term impairment [14]. Even though side effects were deemed a major challenge for compliance by some local actors, during the MDA, only 1.5% of household members refused to initiate ASAQ-CP for fear of side effects, even though, i) they were made aware of possible side effects by CVs, ii) many were familiar with side effects from prior experience, and iii) ASAQ side effects can potentially mimic Ebola symptoms. Also the risk of overdosing appears to have been well understood by the population; one fifth of those not initiating ASAQ-CP in the second round reported starting the first ASAQ-CP course late and needing to wait for one month before beginning the next round of ASAQ-CP.

### Effectiveness of the MDA

The initial self-reported fever incidence in children under five years of age of 6.5% prior to the first round of MDA is low when compared with previously reported estimates of laboratory confirmed malaria prevalence in Monrovia [4]. Underreporting of fever and malaria in our study may have occurred due to stigmatization of Ebola patients and the similarity of malaria and Ebola symptoms. However, given that the first round of MDA was carried out close to the peak of the Ebola outbreak in Monrovia, the bias due to underreporting should be similar or reduced in r2 of MDA compared to r1. Nevertheless, it has been suggested that malaria maybe over-reported in the initial phase of study implementation and that reports decrease over time, possibly because respondents hope to receive more support with higher burden of disease initially [15]. However, the significant difference self-reported fever reduction for household members completing ASAQ-CP and those that did not would not be explained by the phenomenon of systematic under- or over-reporting.

The reduction in fever incidence among those completing the full course of ASAQ in the first round was significantly larger compared to those not initiating or not completing the ASAQ course. This decrease in incidence is coherent with previous knowledge as ASAQ has not been used in MDAs for chemoprevention previously but proved efficient as treatment in Liberia [13] and previous MDAs of malaria chemoprevention have been shown to reduce malaria morbidity over limited time periods [8, 10, 11, 16, 17] tional vector control measures by non-governmental or governmental organizations were not carried out in the distribution area during our intervention. Seasonal changes between the first round in October/November and the second round in November/December may have contributed to the reduction of fevers but cannot explain the significant difference between those that completed ASAQ-CP and those that did not.

With the observed adherence to a full course of ASAQ-CP of 48% in the first round, extrapolation of the overall reduction in incidence of self-reported fever from 4.2% to 1.5% in the sample to the population of the target zone (551,971 individuals) suggests that 14,821 (95% CI 4,801–24,840) fever episodes per month may have been averted as a result of the MDA in Monrovia. Mathematical modelling conducted in early 2015 suggested that in the absence of adequate healthcare provision, three rounds of ASAQ-MDA with coverage of 70% in the whole of Liberia from January 2015 would have had averted 300,000 to 700,000 malaria cases [18]. Given that this MDA targeted about 10% of the Liberian population and consisted of two rounds, our estimates are consistent with modelling results [18]–and indicate that self-reported fever is a good proxy for malaria in this particular setting.

In addition to the reduction in incidence of self-reported fever, the availability of left over ASAQ likely resulted in a reduction of movements to search for malaria treatment and may have reduced transmission of communicable diseases in health facilities.

### Limitations of the evaluation

Potential selection biases may have occurred and affected the findings. First, our sample was based on households selected during voucher distribution, and completeness of voucher distribution could not be concluded from the data available. Therefore, no inference about coverage in the target area can be made. Second, 20% of the target community was included in the sampling frame for the pilot evaluation and could not be included in this evaluation. Third, 39% of sampled households were not reached in both rounds using the phone number provided. We do not know if those individuals that were not reached after three attempts differed systematically from those that were reached in both rounds. Comparing households that were reached in only one round with those reached in both, did not indicate differences in household composition, self-reported side effects or fever incidence. Nevertheless, there is possible sampling bias introduced by those households that were not reached in any of the rounds as they might have a more critical attitude towards the MDA and did not answer the phone for that reason. Additionally, individuals that did not respond may have died or lived in very remote corners of the MDA area without stable phone connections and might differ in other characteristics as well. Unfortunately, it was not possible to train all CVs in basic data collection and the no-touch policy heavily restricted opportunities for direct interaction with the community, so no background information on sampled but not reached households could be obtained. On the other hand, CVs reported that all selected households agreed to participate and provided a phone number. This indicates a) high acceptability of the phone survey and b) a sufficiently good coverage of phone ownership even in disadvantaged areas of Monrovia.

Reporting bias may have been introduced as the prevalence of malaria parasitemia could not be established and self-reported episodes of fever were used as a proxy for malaria, leading to possible estimation-bias of malaria incidence, particularly in the first round [15]. Fever might be under-reported for fear of stigma or over-reported with the hope of receiving additional support. In addition, fever may be caused by other illnesses than malaria. However, the aim of the intervention was to reduce the number of fever cases in Monrovia; therefore, we believe, that the low specificity of fever as a symptom did not negatively impact the ability of the study to measure the effectiveness of the intervention. In addition, the coherence of our results with previous modelling results for malaria incidence indicates that fever may actually be an acceptable proxy for malaria in this context [18].

Lastly, this analysis does not include fever incidence figures from non-MDA areas. It is therefore difficult to determine whether the observed differences in reported incidence were exclusively attributable to the intervention.

## Conclusion

The apparent reduction in self-reported fever cases following the MDA of malaria chemoprevention suggests that the intervention may have been effective in reducing the number of fever cases during the Ebola outbreak in Monrovia. Despite high acceptance and coverage of the MDA and the small impact of side effects, initiation of malaria chemoprevention was low, possibly due to health messaging and behaviour in the pre-Ebola outbreak period and the ongoing lack of healthcare services. Stronger community involvement by dedicated local staff prior to the intervention and improved coordination with other actors in the target area may have been helpful in identifying relevant sensitization messages and hidden communities earlier, and could have possibly addressed misconceptions, fears and rumours more effectively. Combining MDAs during Ebola outbreaks with longer term interventions to prevent malaria and to improve access to healthcare might reduce the proportion of respondents saving their treatment for future malaria episodes.
